# Correlation of FGF-23 With Biochemical Markers and Bone Density in Chronic Kidney Disease-Bone Mineral Density Disorder

**DOI:** 10.7759/cureus.33879

**Published:** 2023-01-17

**Authors:** Imran Hussain, Rishman Tandi, Gurpreet Singh, Gurnoor Kaur, Abhishek ., Saikrishna Dodda, Dirgha Patel, Balaganesh Natarajan, Tejaswini Maram, Ansh Kedia, Roopeessh Vempati, Sweta Sahu, Udit Choubey

**Affiliations:** 1 Nephrology, Osmania Medical College, Hyderabad, IND; 2 Medicine, Government Medical College Amritsar, Amritsar, IND; 3 Medicine, Navodaya Medical College, Raichur, IND; 4 Medicine and Surgery, Self Employed Medical Officer, Vadodara, IND; 5 Medicine and Surgery, Medical College Baroda, Vadodara, IND; 6 Neurology, St. George's University School of Medicine, St. George's, GRD; 7 Medicine, Mahadevappa Rampure Medical College, Gulbarga, IND; 8 Modern Medicine, Institute of Medical Sciences, Banaras Hindu University, Varanasi, IND; 9 Internal Medicine, Gandhi Medical College & Hospital, Hyderabad, IND; 10 Surgery, Jagadguru Jayadeva Murugarajendra (JJM) Medical College, Davanagere, IND; 11 Medicine, Shyam Shah Medical College, Rewa, IND

**Keywords:** hd ( hemodialysis ), chronic kidney disease-mineral and bone disorder, chronic kidney disease, bone mineralisation, fgf 23

## Abstract

Aim

The purpose of this study was to determine the relationship between biochemical markers such as serum calcium (Ca), phosphorus (P), intact parathyroid hormone (iPTH), 25(OH) vitamin D, and fibroblast growth factor 23 (FGF23) in our study group, as well as to correlate dual-energy X-ray absorptiometry (DEXA) findings with these biochemical markers.

Methodology

An eligible group of 50 chronic hemodialysis (HD) patients, age 18 and older, who have undergone HD two times a week for at least six months participated in this retrospective cross-sectional study. We compared serum FGF23, intact parathyroid hormone (iPTH), 25(OH) vitamin D, calcium, phosphorus, and dual-energy X-ray absorptiometry scan showing bone mineral density disorder (BMD) around the femoral neck, distal radius, and lumbar spine. Human FGF23 Enzyme Linked Immuno Sorbent Assay (ELISA) Kit PicoKine® (Catalog # EK0759; Boster Biological Technology, Pleasanton, CA) was used in the optimum moisture content (OMC) lab to measure FGF23 levels. For the analysis of associations with various studied variables, the levels of FGF23 were split into two groups, which were high (group 1, FGF23 50 to 500 pg/ml), that is, up to 10 times the normal levels and extremely high (group 2, FGF23 > 500 pg/ml) FGF23 levels. All the tests were conducted for routine examination where the data obtained was analyzed in this research project.

Results

The mean age of patients was 39.18 ±12.84 years, of whom 35 (70%) were males and 15 (30%) were females. For the entire cohort, serum PTH levels were consistently high, and vitamin D levels were low. FGF23 levels were high in the whole cohort. The average iPTH concentration was 304.20 ± 113.18 pg/ml, while the average 25(OH) vitamin D concentration was 19.68±7.49 ng/ml. The mean FGF23 levels were 1877.36±1378.67 pg./ml. The mean calcium value was 8.23±1.05 mg /dl and the mean phosphate of 6.56±2.28 mg /dl. In the whole cohort, FGF23 showed a negative correlation with vitamin D and a positive correlation with PTH, but not statistically significant. Extremely high FGF23 levels were associated with lower bone density compared to high FGF23 values. Considering that in the whole cohort of patients, only nine had high FGF-23 and the rest of 41 patients had extremely high FGF23, we could not ascertain differences in PTH, calcium, phosphorus, and 25(OH) vitamin D levels between the two groups. The average length of time on dialysis was eight months, and there was no link between FGF-23 levels and the length of time on dialysis.

Conclusion

Bone demineralization and biochemical abnormalities are a hallmark in chronic kidney disease (CKD) patients. Abnormalities in serum phosphate, parathyroid hormone, calcium, and 25(OH) vitamin D play critical roles in the development of BMD in CKD patients. With the discovery of FGF-23 as a biomarker that is increased early in CKD patients, new questions arise about the effects and actions of FGF-23 in controlling bone demineralization and other biochemical markers. Our study found no statistically significant correlation to suggest an effect of FGF-23 on these parameters. But the findings need to be looked at more in prospective, controlled research, especially to find out if therapies that successfully target FGF-23 can make a big difference in how people with CKD feel about their health.

## Introduction

Phosphoprotein fibroblast growth factor 23 (FGF-23) has recently been discovered to regulate phosphate metabolism. It produces phosphaturia and lowers plasma 1,25(OH)2-vitamin D3 levels. It regulates vitamin D and phosphate homeostasis independently of the calcium-parathyroid hormone (PTH)-vitamin D axis. We previously understood bone metabolism based on the vitamin D-PTH-calcium axis. The primary stimulator of PTH production is low calcium in parathyroid cells. The parathyroid hormone increases 1,25(OH)2-vitamin D3 concentration in the blood by stimulating kidney production of 1-alpha hydroxylase. 1,25(OH)2-vitamin D3 boosts renal calcium reabsorption. In response to increased 1,25(OH)2-vitamin D3 levels, intestinal calcium absorption and bone phosphate and calcium outflow are enhanced. The secondary effects of high phosphate levels can be mitigated, thanks to PTH, which stimulates the excretion of phosphate. When calcium and phosphate levels need to be maintained despite an illness like renal failure, PTH levels rise and 1,25(OH)2-vitamin D3 levels fall. As renal failure progresses, these systems begin to fail [1].

FGF-23, originally isolated from the ventrolateral thalamus of the mouse brain [2], is primarily produced by osteoblasts and osteocytes in bone, the thymus, and lymph nodes. High levels of FGF-23 have been linked to lower levels of 1,25(OH)2-vitamin D3 in X-linked hypophosphatemic rickets, autosomal dominant/recessive hypophosphatemic rickets, and tumor-induced osteomalacia. In vivo, hyperphosphatemia and normal 1,25(OH)2-vitamin D3 levels are achieved by the administration of antibodies blocking FGF-23, which reduces urinary fractional excretion of phosphate (UFE phosphate). Prior to treatment with FGF-23 antibodies, moderate chronic kidney disease (CKD) rats with normal serum phosphate levels had high UFE phosphate levels and low 1,25(OH)2-vitamin D3 levels. These results indicate that FGF-23 is necessary for phosphate and vitamin D level changes. Also, the fact that FGF-23 levels rise in early CKD without PTH or phosphate issues lends credence to the theory that FGF-23 maintains healthy phosphate levels [3]. There is a lack of consensus on whether or not phosphate in the diet plays a role in stimulating FGF-23 production. Larsson et al. fed six healthy males with normal renal function a diet supplemented with phosphate. Four times a day, blood samples were taken to check phosphorus and FGF-23 levels. There was no evidence of circadian variation in FGF-23 levels, which were within the normal range. There was no change in blood levels of phosphate, 1,25(OH)2-vitamin D3, or parathyroid hormone. 24-hour phosphate loading increased excretion but did not affect FGF-23 levels [4]. In contrast to the previous study, a phosphate-limited diet decreased FGF-23 concentrations and increased 1,25(OH)2-vitamin D3 concentrations in 13 healthy people. FGF-23 levels were found to be inversely related to 1,25(OH)2-vitamin D3 levels [5]. As FGF-23 increases phosphaturia while keeping serum vitamin D levels constant, it appears to be a hormone that acts in opposition to vitamin D. As nephrons fail, there is a concomitant decrease in the excretion of phosphate and in FGF-23's ability to do so. FGF-23 levels rise gradually, often by a factor of 1,000, in end-stage renal disease (ESRD) to keep phosphate homeostasis stable. There may be a direct link between FGF-23 and PTH, but the exact mechanism still remains unclear.

The Accelerated Mortality on Renal Replacement (ArMORR) study used a dataset of 1056 US dialysis sites to investigate the link between FGF-23 and mortality in hemodialysis patients. Prospectively, data from 10,044 people who began hemodialysis and were followed for a year were analyzed. After removing patients exposed to vitamin D at the entry point, inactive C-terminal FGF-23 (cFGF-23) levels were measured along with full-length intact FGF-23 (iFGF-23) levels. The association between iFGF-23 and cFGF-23 was found to be linear. These models considered baseline demographics, primary renal diagnosis, associated comorbidities, coexisting conditions, dialysis dose, and facility-specific standardized mortality rates. High cFGF-23 levels were strongly linked to death, regardless of phosphate or other known risk factors. The relationship of FGF-23 and mortality was dose-responsive and seen on a continuous scale (odds ratio 1.8; 95% CI, 1.4 - 2.4 per unit increase in log cFGF-23) and for each quartile (quartile 1 served as 67 reference and odds ratio for quartile 2, 3 and 4 were 1.6 {95% CI, 0.8 - 3.3}; 4.5 {95% CI, 2.2 - 9.4}; and 5.7 {95% CI, 2.6 to 12.6} respectively) [6]. A secondary analysis of The Evaluation Of Cinacalcet Hydrochloride (HCl) Therapy to Lower CardioVascular Events (EVOLVE), a randomized, prospective, multicenter placebo-controlled trial, revealed that cinacalcet therapy significantly reduces FGF-23. Ninety-one percent of the patients had blood samples taken at baseline and after 20 weeks while staying on the original medication. At baseline, 2985 (77%) of the 3883 randomized patients had FGF-23 and 2602 (67%) had both baseline and week 20 results available. Luminex-based microbead assays using polyclonal antibodies were used to analyze samples. The median FGF-23 levels at baseline were comparable (cinacalcet group, 5555 pg/ml; placebo, 5600 pg/ml; P = 0.86). In the placebo group, FGF-23 levels were significantly lower than those in the cinacalcet group (5580 pg/ml) at week 20. The key endpoints were tracked for five years, and FGF-23 levels were linked to these events. GFGF-23 reductions were linked to fewer cardiovascular events and fatalities [7]. There is evidence that high levels of FGF-23 are linked to left ventricular hypertrophy (LVH), and cinacalcet lowers calcium levels, but it is impossible to say that lower levels of FGF-23 cause fewer cardiovascular events.

## Materials and methods

This is a retrospective study done on patients who underwent hemodialysis (HD). About 50 patients over 18 years old who underwent HD at least twice a week for at least six months were recruited in this study. FGF-23, intact parathyroid hormone (iPTH), 25(OH) vitamin D, calcium, and phosphorus levels measured in the serum as well as bone mineral density disorder (BMD) measured by dual-energy X-ray absorptiometry (DEXA) at the femoral neck, distal radius, and lumbar spine during workup, were considered as the study parameters. Patients with pre-existing primary parathyroid abnormalities, on non-steroidal anti-inflammatory drugs (NSAIDs), with known liver disease, who had received steroids, with automated external defibrillators (AEDs), on HD for the past six months, who were dialysis-dependent on continuous ambulatory peritoneal dialysis (CAPD), and who were non-dialysis dependent CKD patients were excluded from the study.

Vitamin D

Vitamin D in its 1,25-hydroxylated form is the active form that has been shown to have beneficial effects. However, its half-life in the bloodstream is very short. Hence, its metabolite 25-hydroxy vitamin D (25-OH vitamin D) is used to assess the body's overall vitamin D status. 25-OH Vitamin D has a half-life of two to three weeks and is the major storage form. The kit used to measure this parameter was ms-05894913 190 (Roche Products, Mumbai, India) and was carried out on Cobas e411 ECLIA analyzer (Roche diagnostics, Rotkreuz, Switzerland). The stability of vitamin D in K2 EDTA (dipotassium ethylenediamine tetraacetic acid) plasma at -20°C is for 24 weeks. The measuring range is 3.00-70.0 ng/ml. 

PTH

The 2-site ELISA was used for the iPTH assay. On Cobas e 411 analyzers, the kit used is ms-11972103122. The antibodies used in this test bind to the N-terminal 1-37 amino acid epitope at 26-32 amino acid epitope and the C-terminal 38-84 amino acid epitope at 37-42 amino acid epitope. 1.20-5000pg/ml is the measuring range. PTH is stable in plasma at -20°C for 6 months. This method does not react with PTH 1-37 or parathormone-related peptide (PTHrP) 1-86. The shelf life of an unopened kit at 2-8°​​C is up to the expiry date and for an opened kit the shelf life at 2-8°​​C is 12 weeks, and on the analyzer, it is eight weeks.

Calcium

The Calcium Gen 2 kit-05061482 190 (Roche Products, Mumbai, India) is used to conduct this assay, and Cobas c 311 analyzers were utilized to conduct the analysis. The variation in absorbance of light with a wavelength of 376 nm, which is measured photometrically, serves as the basis for the test. At a temperature of -20°​C, the serum calcium has an eight-month shelf life. This method has a measurement range of 0.8-20.1 mg/dl as its possible output. The total calcium concentration in the serum is the parameter that is measured. When calculating corrected calcium, it is adjusted so that it takes serum albumin into account.

Phosphorus

This test is done on Cobas c 311 analyzers with the inorganic phosphate ver.2 kit-03183793-122 (Roche Products, Mumbai, India). The test is based on the molybdate UV principle, which measures the change in absorbance of ammonium phosphomolybdate, which breaks down into molybdenum. The phosphate ions in serum kept at -20°​C are stable for a year.

FGF23

A solid phase assay PicoKine® human FGF23 precoated ELISA kit (Catalog # EK0759; Boster Biological Technology, Pleasanton, CA) was used to measure FGF23. The normal range for adults (> or =18 yrs): < or = 59 pg/mL

BMD by DEXA

DEXA scans were performed using a GE Lunar-1 Co prototype machine (GE Medical Systems Lunar, Madison, Wisconsin). The World Health Organization's (WHO) definition of osteoporosis served as the basis for both the DEXA definition and the bone mass criteria used to diagnose the disease (1994). According to the World Health Organization's definition of osteoporosis, the T score was used to evaluate BMD and define different stages of BMD. T measurements were obtained from the femoral neck, L1, L2 (L- lumbar), and the distal radius. Osteoporosis was defined as a T-score lower than -2.5 SD. T scores between -1 SD and -2.5 SD were considered to indicate osteopenia, while T scores above -1 SD were considered to be within the normal range.

Method of data collection

For routine follow-up blood investigations, approximately 8 mL of venous blood, was drawn from the median cubital vein, with the patient's agreement. All samples were obtained in accordance with the instructions. From the collected sample, 3 mL of blood was collected in the K2 EDTA tube for plasma separation. In the typical serum-separating tube with a red cap, 5 mL of blood was collected. After approximately one hour of clotting at room temperature, samples were centrifuged and serum/plasma was separated. Serum and plasma samples were then transferred to be stored in ice-lined coolers at -20°​C. All specimens were examined within three months of collection. Serum and plasma samples were tested for all other biochemical markers, including 25(OH) vitamin D and iPTH. FGF-23 was measured using the boster picoline human FGF-23 precoated ELISA kit EK0759.

Statistical analysis

Version 18 of the SPSS statistical software (SPSS Inc., Chicago) was used to analyze the data. The Pearson chi-square test, the independent sample t-test, and the Pearson correlation were utilized to establish the association between various factors and correlation with data. The criterion for statistical significance was a P value of 0.05.

## Results

There were a total of 50 participants, with 35 men and 15 women. Twenty-five patients were older than 40 years, while 25 patients were younger than 40 years. It was revealed that the mean age of patients was 39.18 ±12.84 with a median age of 39 years. The mean height was 166.86 cm ± 6.92 and the mean weight of 52.50± 8.83 Kg. In our study, the mean serum calcium was 8.23mg/dl ±1.05mg/dl, and the mean phosphate was 6.56mg/dl ± 2.28mg/dl (Table 1). 

**Table 1:** Characteristics of the study subjects

Variable
Age (years)
Mean 39.18±12.84
Median 39(28=47)
Gender-n (%)
Male 35(70)
Female 15(30)
Height (cm)
Mean 166.86±6.92
Median 166.50(162-172)
Weight (kg)
Mean 52.50±8.83
Median 50.50(46-56.25)
Serum calcium (mg/dl)
Mean 8.23±1.05
Median 8.05(7.70-8.85)
Serum phosphate (mg/dl)
Mean 6.56±2.28
Median 6.85(4.5-8.12)
Parathyroid Hormone (pg/ml)
Mean 304.20±113.18
Median 290.50(222.75-367.25)
Fibroblast growth factor 23 (pg/ml)
Mean 1877.36±1378.67
Median 1782.50(709.75-2875.75)
25(OH) Vitamin D (ng/ml)
Mean 19.68±7.49
Median 21(12.75-24.50)

The bone mineral density test was done at the femoral neck, distal radius, and L1, L2, and results were obtained. At the femoral neck, 30% of patients were osteoporotic (n = 15), 48% were osteopenic (n = 24), and 22% were normal (n = 11) (Table 2). 

**Table 2:** Bone mineral density test by DEXA at femoral neck DEXA: Dual-energy X-ray absorptiometry

Femoral Neck	Frequency	Percentage
Osteoporosis	15	30
Osteopenia	24	48
Normal	11	22

At the distal radius, 28% of patients (n = 14) were osteoporotic, 42% were osteopenic (n = 21), and 30% had normal bone mineral density by dual-energy x-ray absorptiometry (n = 15) (Table 3). 

**Table 3:** Bone mineral density test by DEXA at distal radius DEXA: Dual-energy X-ray absorptiometry

DEXA Scan distal radius T score	Frequency	Percentage
Osteoporosis	14	28
Osteopenia	21	42
Normal	15	30

In the lumbar spine, 28% of patients (n = 14) were osteoporotic, 40% were osteopenic (n = 20), and 32% had normal bone mineral density by DEXA (n = 16) (Table 4).

**Table 4:** Bone mineral density by DEXA at lumbar spine level DEXA: Dual-energy X-ray absorptiometry

Dexa Scan L1 T score	Frequency	Percentage
Osteoporosis	14	28
Osteopenia	20	40
Normal	16	32

Low bone mineral density at femoral neck was associated with higher mean age, higher mean serum phosphate, higher mean serum FGF23 levels, and a lower mean serum 25(OH) vitamin D although none of the associations was statistically significant (Table 5).

**Table 5:** Association of osteoporosis with various mean variables

Variable	Osteoporosis		P value
	Present	Absent	
Age (years)			
Mean ± SD	42.87±14.60	37.60±11.89	0.231
Sex			
Male	10(66.7)	25(71.4)	0.140
Female	6(46.2)	9(24.3)	0.140
Calcium (mg/dl)			
Mean ± SD	8.02±1.00	8.31±1.08	0.369
Phosphate (mg/dl)			
Mean ± SD	7.17±1.74	6.30±2.45	0.163
Parathyroid Hormone (pg/ml)			
Mean ± SD	299.17±125.93	306.40±109.15	0.846
FGF-23 (pg/ml)			
Mean ± SD	2166.20±1593.39	11753.57±1281.12	0.888
25(OH) Vitamin D (ng/dl)			
Mean ± SD	17.33±7.49	20.69±7.37	0.157

**Figure 1:**
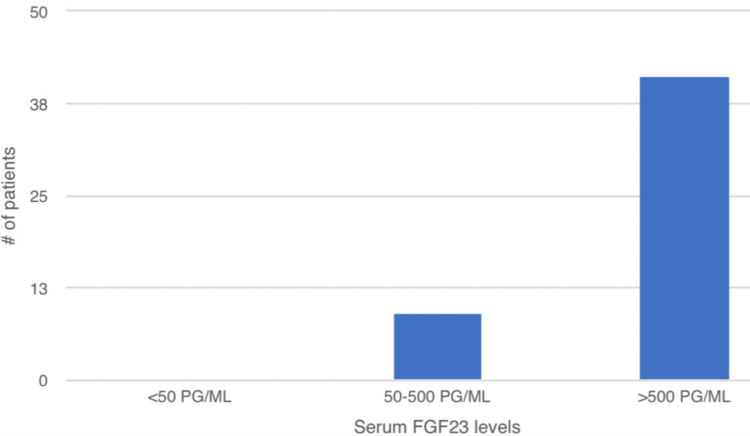
Distribution of serum FGF-23 in the study population

The mean FGF-23 was 1877.36 pg/ml ± 1378.67pg/ml. FGF-23 levels were between 50 and 500 pg/ml in 18% (n = 9), over 500 pg/ml in 82% (n = 41), and less than 50 pg/ml in none of the patients (Figure 1).

The mean serum FGF-23 was 1877.36 pg/ml ± 1378.67pg/ml. Around 18% had values up to 5-fold above normal, and 82% had levels above 5-to-10-fold normal values (< or = 59 pg/mL). As FGF-23 levels start rising early in CKD as compensation to prevent hyperphosphatemia, these high values of FGF-23 were consistent with other studies. There was a negative correlation between FGF-23 and 25 (OH) vitamin D. Serum phosphate was positively correlated with serum PTH and FGF-23, with P values being statistically significant between serum phosphate and serum PTH. Serum phosphate was also negatively correlated with serum 25(OH) vitamin D (Table 6).

**Table 6:** Correlation of serum phosphate with other biochemical parameters PTH: Parathyroid hormone; FGF23: Fibroblast growth factor-23

Variable	Correlation coefficient	P value
PTH	0.284	0.045
FGF23	0.134	0.354
25(OH) Vitamin D	-0.141	0.329

The correlation was negative between PTH and serum 25(OH) vitamin D and P values were significant. The correlation between serum PTH and FGF-23 was positive but did not reach statistical significance (Table 7).

**Table 7:** Correlation between serum PTH and other biochemical markers FGF23: Fibroblast growth factor-23

Variable	Correlation coefficient	P value
25(OH) Vitamin D	-0.28	0.049
FGF23	0.223	0.119

The calcium values were negatively correlated with serum phosphate and serum PTH, with the P value being statistically significant for the correlation between serum calcium and serum PTH levels. Serum calcium levels were positively correlated with serum FGF-23 and serum 25(OH) vitamin D levels, with the correlation between serum calcium and 25(OH) vitamin D being significant (Table 8). 

**Table 8:** Correlation of serum calcium with other biochemical markers PTH: Parathyroid hormone; FGF23: Fibroblast growth factor-23

Variable	Correlation coefficient	P value
Phosphorous	-0.209	0.145
PTH	-0.283	0.047
FGF23	0.191	0.184
25(OH) Vitamin D	0.292	0.039

**Figure 2:**
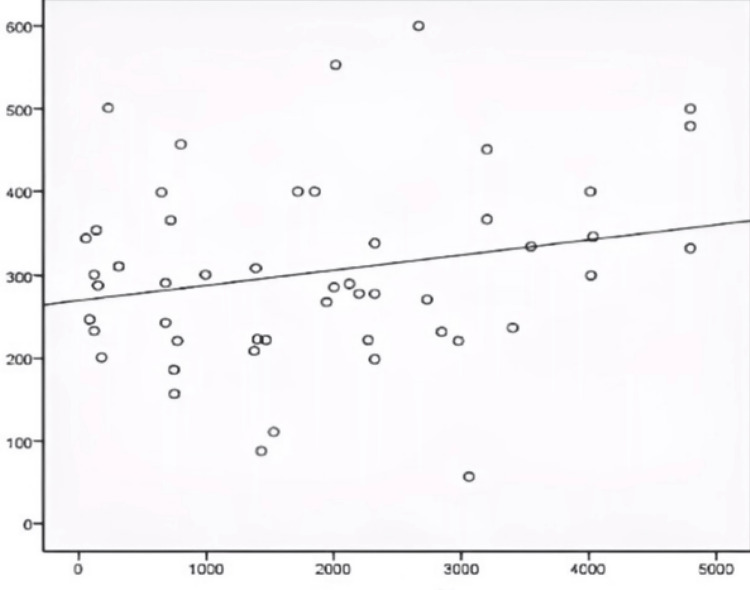
Correlation between FGF-23 and PTH X-axis: FGF-23 (pg/ml), Y-axis: PTH (pg/ml)

There was a noticeable positive correlation between FGF-23 and serum PTH levels (Figure 2).

**Figure 3:**
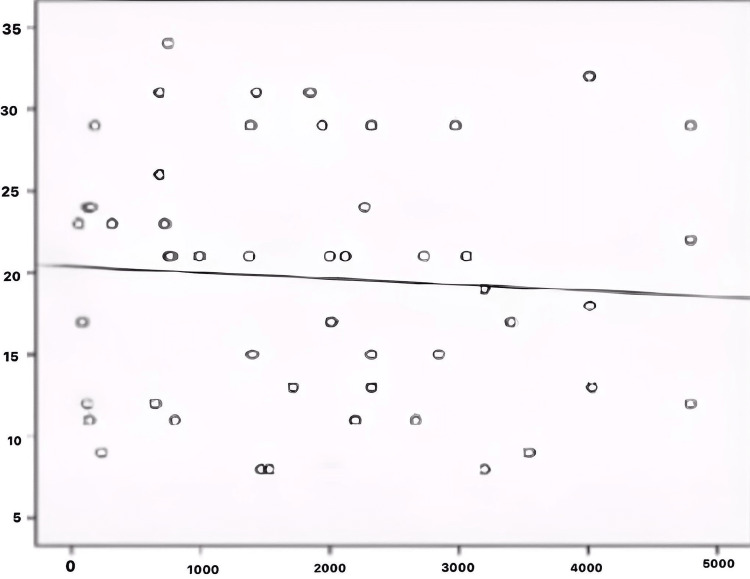
Correlation between FGF-23 and 25(OH) Vitamin D X-axis: FGF-23 (pg/ml), Y-axis: 25(OH) Vitamin D (ng/ml)

There was a noticeable negative correlation between FGF-23 and serum 25 (OH) vitamin D levels (Figure 3).

## Discussion

A study by Anandh et al. in HD patients (n = 91) with a mean dialysis vintage of 47.2 months had a mean FGF-23 of 1152.7 pg/ml [8]. In another study in HD patients by Torres et al. (n = 99) with a mean HD vintage of 6.8 years, the mean FGF-23 was >30000pg/ml [9]. In a study by Zheng et al. (n = 125), the mean dialysis vintage was 65 months, and the mean FGF-23 values were 1020pg/ml [10]. So, by and large, our results were consistent with other national and international studies. Although none of the associations were statistically significant, FGF-23 was positively correlated with serum PTH and phosphate and negatively correlated with serum 25(OH) vitamin D. Both serum FGF-23 and serum PTH rise early in CKD before phosphate rises. In a study by Anandh et al., FGF-23 was significantly associated with serum phosphorus and PTH as in our study [8].

We build on earlier research in this work to show that all CKD and HD patients have incredibly increased serum FGF-23 levels. Dialysis does not remove FGF-23 from the body or lower FGF-23 levels in the serum. To compensate for the drop in phosphate excretion that occurs along with the decline in glomerular filtration, serum FGF-23 levels rise in the early stages of renal illness. When these individuals are treated with hemodialysis for ESRD, their serum FGF-23 levels rise more noticeably than anticipated given their hyperphosphatemia level. Our findings further support that high levels of FGF-23 in serum in HD patients with normal or low serum phosphate concentration cannot be attributed to hyperphosphatemia. Blood FGF-23 levels in these individuals were more than 10 times greater than the increased values seen in the community of healthy people. Only a modest relationship was seen with the serum phosphate levels.

Consequently, in these HD patients with totally impaired renal function, a considerable amount of high serum FGF-23 is most likely due to its buildup in the body due to an insufficiency of renal catabolism or renal clearance. Similar to autonomous hyperparathyroidism, HD patients may have unbalanced FGF-23 synthesis and/or secretion, resulting in high blood FGF-23 levels. Our findings suggest that PTH does not amplify serum FGF-23. Calcitriol increases serum FGF-23 in HD patients, suggesting vitamin D therapy may boost FGF-23 levels. We found a correlation between serum FGF-23 and plasma vitamin D levels, but it was not statistically significant. Fractures and demineralization of bone are common in HD patients. Bone demineralization in individuals who have CKD is caused by a complex and not fully understood mechanism [11-13].

Although high PTH and low plasma 1,25(OH) vitamin D levels contribute to the pathophysiology of this illness, they cannot fully explain it [14]. 25(OH) vitamin D is quite deficient in CKD patients mostly due to reduced reabsorption of it from the proximal collecting tubule. There is also a marked reduction in cutaneous synthesis of 25(OH) vitamin D. High serum FGF-23 levels have now been added to the list of these indicators, which can predict the severity and resistance of secondary hyperparathyroidism in dialysis patients [15,16]. Although an increased level of serum FGF-23 concentrations cannot cause hypophosphatemia in patients undergoing dialysis, it is unknown if FGF-23 buildup can directly influence bone mineralization. At various sites, we discovered a weak or no connection between serum FGF-23 and BMD. Our results demonstrate that FGF-23 does not directly affect the mineralization of the skeletal system in CKD dialysis patients. Our findings are comparable with those of Urena et al., who found no association between serum FGF-23 and BMD [9]. Coskun et al. did a study and got the same results: there was no link between serum FGF-23 and bone-mineral density values in bone disease [17]. 

## Conclusions

Bone demineralization and biochemical abnormalities are hallmarks of CKD patients. Abnormalities in serum phosphate, PTH, calcium, and 25(OH) vitamin D play a critical role in the development of BMD in CKD patients. With the discovery of FGF-23 as a biomarker that is increased early in CKD patients, new questions arise about the effects and actions of FGF-23 in controlling bone demineralization and other biochemical markers. Our study found no statistically significant correlation to suggest an effect of FGF-23 on these parameters. But the findings need to be looked at more in prospective, controlled research, especially to find out if the therapies targeting FGF-23 can make a big difference in how people with CKD feel about their health.
